# Utilization of insecticide-treated bed nets and care-seeking for fever and its associated socio-demographic and geographical factors among under-five children in different regions: evidence from the Myanmar Demographic and Health Survey, 2015–2016

**DOI:** 10.1186/s12936-019-3088-0

**Published:** 2020-01-06

**Authors:** Kyi Thar Min, Thae Maung Maung, Myo Minn Oo, Tin Oo, Zaw Lin, Aung Thi, Jaya Prasad Tripathy

**Affiliations:** 1 Department of Medical Research, Ministry of Health and Sports, Yangon, Myanmar; 2 International Union Against Tuberculosis and Lung Disease (The Union), Mandalay, Myanmar; 3 Vector Borne Disease Control Programme, Ministry of Health and Sports, Nay Pyi Taw, Myanmar; 4 National Malaria Control Programme, Ministry of Health and Sports, Nay Pyi Taw, Myanmar; 5 International Union Against Tuberculosis and Lung Disease (The Union), South-East Asia Office, New Delhi, India; 6 International Union Against Tuberculosis and Lung Disease (The Union), Paris, France; 7 Present Address: All India Institute of Medical Sciences, Nagpur, India

**Keywords:** Insecticide treated net, Under-five children, Health care seeking, Demographic and Health Survey, SORT IT, Myanmar

## Abstract

**Background:**

Malaria is one of the top-five contributors to under-5 deaths in Myanmar. Use of insecticide-treated nets (ITN) and receiving early appropriate care in case of fever are the core interventions to prevent malaria and its complications and thereby deaths. This study aimed to assess among the under-five children, (a) utilization of ITNs and its associated factors, (b) care-seeking behaviour among their caregivers and its associated factors and uptake of malaria testing among those with fever in the last 2 weeks.

**Methods:**

This was a cross sectional study using secondary analysis of Myanmar Demographic and Health Survey (MDHS) conducted in 2015–2016. Multivariable logistic regression was used to explore the factors associated with non-utilization of ITNs and not seeking care for fever. Effect sizes have been presented using odds ratios with 95% confidence intervals. Data analysis was done using *svyset* command in STATA to account for the multi-stage sampling design of the survey.

**Results:**

Of 4597 alive under-five children, 80.5% did not sleep under an ITN last night. The factors significantly associated with non-utilization of ITNs were residing in malaria elimination regions (aOR = 2.0, 1.3–3.2), urban residence (aOR = 1.8, 1.2–2.9), staying in delta region (aOR = 8.7, 4.7–12.2), hilly region (aOR = 3.0, 2.0–4.6, and having highest wealth quintile (aOR = 1.8, 1.1–3.0). Around 16% had fever in the last 2 weeks, of whom 66.7% sought care for fever and 3% got tested for malaria. Nearly half (50.9%) of the caregivers sought care from a government health facility, followed by private hospital/doctor (27.8%), shop (8.0%), village health worker (4.4%) and pharmacy (3.1%). The factors associated with not seeking care for fever were residing in specific geographical locations (hilly, delta and central plains compared to coastal region) and having lowest wealth quintile (aOR = 2.3, 1.1–5.7).

**Conclusions:**

This study highlighted that ownership and utilization of ITNs was very poor among under-5children. Care-seeking behaviour of the caregivers of under-5 children in case of fever was dismal with two-thirds not seeking care. The programme should seriously consider addressing these barriers if Myanmar is to achieve zero malaria deaths by 2030.

## Background

Globally, malaria is still an important public health problem with 219 million cases and 435,000 malaria-related deaths worldwide in 2017 [1]. Malaria remains a major killer of under-fives, claiming the life of one child every 2 min. It is the one of the ten leading causes of under-five mortality globally [2].

Under-5 malaria deaths have to be tackled through already known effective preventive and curative interventions. One of the core interventions recommended by the World Health Organization (WHO) to reduce under-5 deaths is sleeping under a bed net. Bed nets are broadly of two types: untreated and treated, which are referred to as insecticide-treated net (ITN)/long-lasting insecticidal net (LLIN), hereafter referred to as ITN [3]. Bed nets treated with an insecticide are much more protective than untreated nets. These insecticides kill mosquitoes as well as other insects. They also repel mosquitoes reducing the number that enter the house. National malaria control programmes across the globe distribute ITNs free of cost to improve ownership and utilization of ITNs. Bed nets have been shown to reduce under-five mortality by 18.8% in Ghana [4] and by 55% in combination with indoor residual spraying [5]. However, poor utilization of ITNs is a major concern, as reported in several studies from Africa [6, 7].

Myanmar has a high burden of malaria with more than two-third of its population at risk of malaria [8]. Malaria is endemic in 291 out of 330 townships in the country. It is also one of the countries in the Greater Mekong Sub-region (GMS) with evidence of artemisinin resistance [9]. However, the National Malaria Control Programme (NMCP) is committed to eliminate malaria from the entire country by 2030 [10].

Under-five children are the most vulnerable group affected by malaria. Malaria is one of the major causes of under-5 death in the country, after prematurity, low birth weight and acute respiratory tract infections [11]. In order to tackle malaria deaths, utilization of ITNs/LLINs in areas of high malaria transmission is one of the key preventive interventions for malaria elimination in Myanmar in all age groups including under-5 children.

Myanmar aims at achieving and maintaining 100% access and utilization of ITNs at the household level. Previous studies from Myanmar have assessed ownership and utilization of ITNs in the general population [12], in specific regions/settings [13, 14] and key migrant occupations such as plantation workers [15, 16]. However, information on ITN usage among under-five children and the associated factors is scarce.

Besides ITN use, the WHO has also emphasized early diagnosis and prompt treatment within 24 h of onset of symptoms to decrease risk of severe complications and death due to malaria [17]. Malaria testing and treatment facilities are available at all public health facilities and at the community level with the village health volunteers in Myanmar and are offered free of cost. Despite this, timely testing and early diagnosis of malaria remains a concern in Myanmar and globally. This requires an understanding of the health-seeking behaviour in case of fever. Previous studies in Myanmar have explored treatment-seeking behaviour of persons with fever in the general population [18] and amongst migrant workers [19, 20]. There is little information regarding care-seeking for fever and uptake of malaria testing services among caregivers of under-five children in Myanmar and its associated factors. A study regarding the treatment-seeking behaviour of caregivers of under-five children in Myanmar was done in a single township, which cannot be generalized to represent the whole country [21].

The first Myanmar Demographic and Health Survey (MDHS) was implemented by the Ministry of Health and Sports (MOHS) in 2015–2016. This nation-wide survey collected useful information on utilization of ITNs and health-seeking behaviour in case of fever [22]. The survey also collected socio-demographic and geographical information about the respondents. These factors could be associated with ITN use and care-seeking behaviour, though the survey did not report these associations. To fill the gaps in literature in Myanmar as mentioned earlier, the present study analyzed the DHS dataset to assess among the under-five children: i. utilization of ITNs and its associated socio-demographic and geographical factors, ii. care-seeking behaviour (number and proportion who sought care for fever and type of first provider sought) and its associated socio-demographic and demographic factors, and iii. uptake of malaria testing among those with fever in the last 2 weeks.

## Methods

### Setting

Myanmar is a south-east Asian country inhabited by 51 million people, 70% residing in rural areas. It is divided into seven states, seven regions and one capital territory (Nay Pyi Taw Council territory) which are further subdivided into 74 districts with 330 townships [8]. The terrain, sub-tropical climate, precipitation and vegetation favours mosquito breeding and malaria transmission.

### Study design

This study analysed secondary data from the MDHS 2015–2016, which is a cross-sectional survey. The details about the survey and its methodology are described below in brief.

### Myanmar 2015–2016 Demographic and Health Survey (DHS)

The Myanmar DHS is a nationally representative survey of women and men aged 15–49 years to assess the population and health status of adults of reproductive age and their children under 5 years. This is part of the worldwide DHS Program which is funded by the United States as well as many other donors. The 2015–2016 survey is the first DHS ever conducted in Myanmar, and was supported by United States Agency for International Development and the Three Millennium Development Goal (3MDG) Fund.

### Sample and sampling technique

**Fig. 1:**
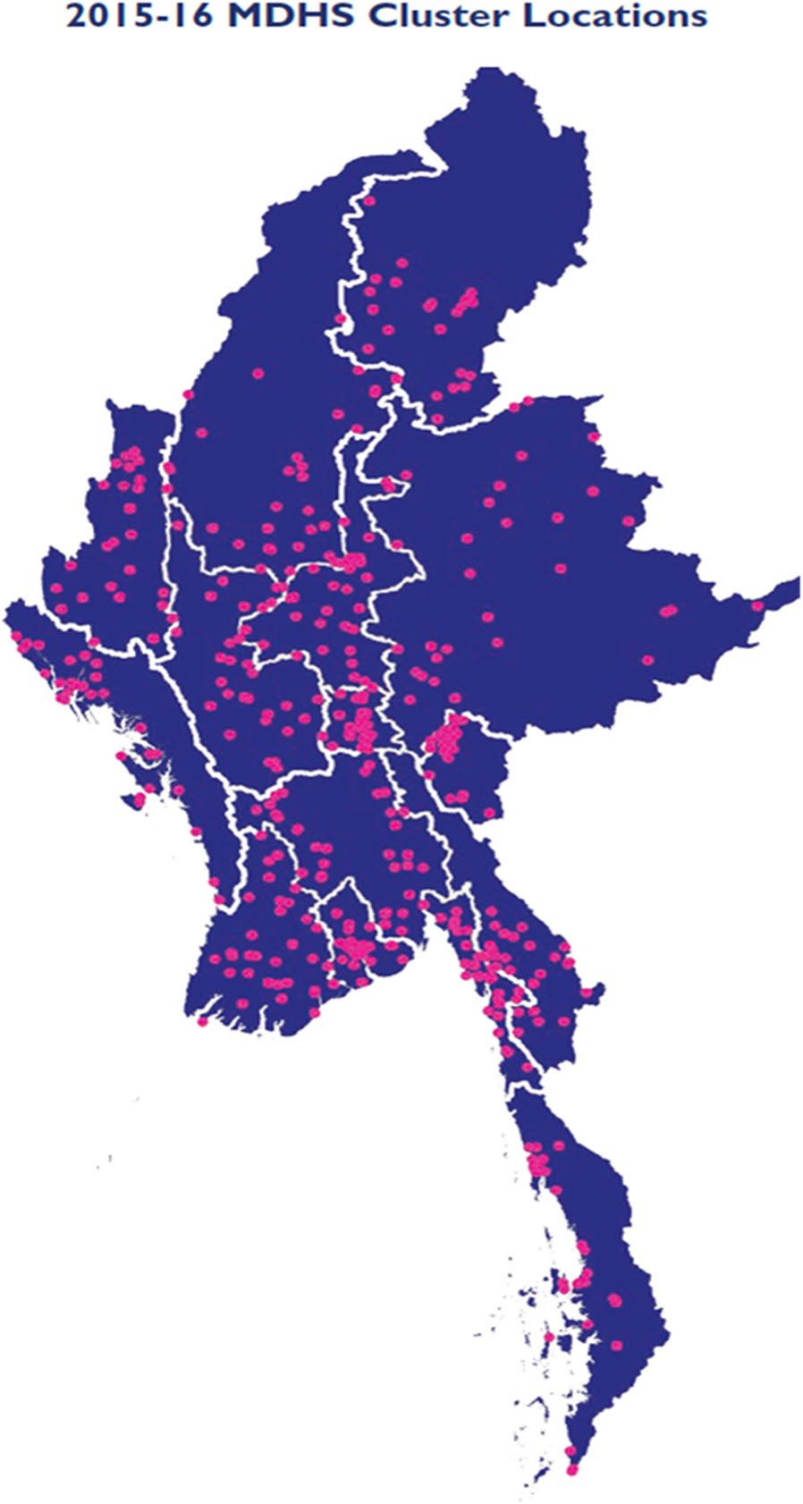
Map showing the clusters covered under the Myanmar Demographic and Health Survey, 2015–2016

The DHS sample has characteristics similar to that of the general population as it covered all states/regions in the country. A master sampling frame was created based on the 2014 census which consisted of 4000 Primary Sampling Units (PSUs) drawn from the entire census frame. Each PSU is either a census enumeration area (EA) or ward in urban areas or village tract in rural areas. The survey followed a stratified two-stage sample design and was intended to allow estimates of key indicators at the national level, in urban and rural areas, and for each of the seven states and eight regions of Myanmar. Stratification was achieved by separating each state or region (n = 15) into urban and rural areas, each of which formed a separate sampling stratum. In total, 30 sampling strata (15*2) were created. The first stage involved selecting clusters/PSUs from these strata. A total of 442 clusters (123 urban and 319 rural) were selected from the master sample (Fig. 1). At the second stage, a fixed number of 30 households was selected from each of the selected clusters (a total of 13,260 households), using equal probability systematic sampling. The response rate was 98%.

All women aged 15–49 years who were either permanent residents of the selected households or visitors who stayed in the households the night before the survey were eligible for interview. In the survey, questions related to under-5 children in the household about the ownership and utilization of ITNs and health-seeking behaviour (in case of fever) were asked to the female respondent in the family.

### Data variables

The key outcome variables included in this study were not sleeping under ITN the last night prior to the survey, not seeking care for fever, first place where care was sought for fever and whether tested for malaria. Other socio-demographic characteristics included the caregiver’s (women aged 15–49 years) age, education and occupation, father’s education and occupation, household characteristics, place of residence and region (See Table 1).

**Table 1:** List of variables, variable labels, field type and their categories extracted from the Demographic and Health Survey Myanmar, 2015–2016

Variable name	Variable label	Field type	Value labels
Respsex	Respondent’s sex	Integer	Female Male Not recorded
Respage	Respondent’s age	Integer	
Childsex	Sex of under-five children	Integer	Female Male
Childage	Children’s age in completed months	Integer	
Wealthindex	Wealth index of household	Integer	Poorest Poor Middle Richer Richest
Occumom	Occupation of mother	I	Dependent Casual Staff Migrant Own
Occudata	Occupation of father	I	Dependent Casual Staff Migrant Own Unknown
Edumom	Education of mother	Integer	Literacy Primary Middle Higher Graduated Not recorded
Edudad	Education of father	Integer	Literacy Primary Middle Higher Graduated
Region	Region where for interviewed HH	Interger	Kachin Kayah Kayin Chin Mon Delta Rakhine Shan Yangon Mandalay Sagaing Magway Bago Thanitarry NayPyiTaw territory
Resid	Place residence of HH	I	Rural Urban
Hhmember	Number of HH member	I	
Numu5	Number of Under 5 children	I	
Sleepu5	Number of U5 children slept under INT	I	Yes No
Hhitn	HH possess ITN	I	Yes No
Feveru5	Number of U5 Child had fever within 2 weeks	I	
U5seek	Number of U5 children with fever taken to health facility or health care provider	I	

### Operational definitions

The care-seeking for fever among under-5 children means seeking advice or treatment from a health provider, a health facility or a pharmacy. This definition is according to the survey guidelines.

An ITN is defined as 1. A factory-treated net that does not require any further treatment (long-lasting insecticidal net, or LLIN) or 2. A pretreated net obtained within the past 12 months or 3. A net that has been soaked with insecticide within the past 12 months.

### Data analysis

Myanmar DHS Data set (Stata file) was analysed using STATA version 15.0. The children (KR) data set from DHS data set was used and a subset of the data related to alive under-5 children was extracted for this study. Key indicators such as ITN utilization and treatment-seeking behaviour for fever within last 2 weeks among under-five children have been presented in the form of proportions (95% confidence intervals). To describe the factors associated with non-utilization of ITN and not seeking care for fever, logistic regression was run and effect sizes have been presented using odds ratios with 95% confidence intervals and p-values. A *p* value less than < 0.05 was considered as significant. The analysis was weighted for the multi-stage sampling design and hence, weighted estimates have been provided. “Svyset*”* command was used in STATA to account for sampling weights, clustering and stratification in the KR dataset.

### Ethics

Ethical approval for this study was obtained from Ethics Review Committee of the Department of Medical Research, Ministry of Health and Sports, Myanmar (Ethic/DMR/2018/164) and The Union Ethics Advisory Group, Paris, France (EAG number: 40/18). Permission to use the DHS data was obtained from the DHS program and the Department of Public Health, Ministry of Health and Sports, Myanmar. This being a secondary data analysis of DHS database, a waiver of informed consent was granted by the ethics committees.

## Results

### Socio-demographic and household characteristics

Of 4815 underweight children who were part of the MDHS, 4597 (95.5%) eligible (alive) children were included in this study. There were no missing data in these children who were part of this study. Of 4597 under-five children, half were males (52%, 2402), predominantly rural residents (77.4%, N = 3617), nearly 40.4% (n = 1842) belonged to 3–5 years age group with almost an equal number in the 1–2 years age group. Around half of the caregivers (45.8%, n = 2034) were educated up to primary level and 55.7% (n = 2425) were working (Table 2).

**Table 2:** Socio-demographic, geographic and household characteristics of under-five children included in Myanmar Demographic and Health Survey, 2015–2016 (N = 4597)

Characteristics	Unweighted count	Weighted %	
Total	N = 4597	(%)	(95% CI)
Age of the child (in months)			
< 12	940	(19.6)	(18.3–21.0)
12–35	1815	(39.8)	(38.3–41.4)
36–59	1842	(40.4)	(38.8–42.0)
Sex of the child			
Male	2402	(52.0)	(50.0–53.8)
Female	2195	(48.0)	(46.1–49.9)
Age of the caregiver (in years)			
15–24	865	(19.0)	(17.4–20.8)
25–34	2368	(52.1)	(50.0–54.1)
≥ 35	1364	(28.7)	(27.0–30.5)
Education of the caregiver			
No formal education	802	(17.8)	(15.1–20.7)
Primary	2034	(45.8)	(43.2–48.4)
Secondary	1440	(28.6)	(26.4–31.0)
More than secondary	321	(7.6)	(6.5–8.9)
Father’s education			
No education	792	(17.7)	(15.2–20.5)
Primary	1797	(40.4)	(38.0–42.9)
Secondary	1664	(34.3)	(31.9–36.9)
More than secondary	253	(5.5)	(4.7–6.9)
Don’t know	89	(1.7)	(1.2–2.2)
Occupation of the caregiver			
Working	2425	(55.7)	(53.1–58.3)
Not-working	2170	(44.2)	(41.6–46.8)
Father’s occupation			
Manual worker	2599	(57.5)	(54.2–60.7)
Agricultural	1280	(26.3)	(23.0–29.9)
Others	718	(16.1)	(14.1–18.4)
No of household members			
1–3	450	(11.0)	(9.7–12.5)
4–6	2606	(58.7)	(56.4–60.9)
7–10	1335	(26.4)	(24.4–28.4)
More than 10	206	(3.8)	(2.9–4.9)
Wealth index			
Lowest	1383	(29.5)	(26.7–32.5)
Second	1043	(22.1)	(20.3–24.0)
Middle	826	(16.8)	(15.2–18.6)
Fourth	775	(17.0)	(15.2–19.0)
Highest	570	(14.4)	(12.5–16.5)
Region			
Malaria elimination regions	1497	(42.0)	(39.5–44.6)
Other regions	3100	(57.9)	(55.3–60.4)
Geographical location			
Hilly	1810	(23.3)	(21.2–25.6)
Coastal	895	(13.6)	(11.9–15.4)
Delta	810	(32.3)	(29.8–34.8)
Central plains	1082	(30.6)	(28.3–33.1)
Place of residence			
Urban	980	(22.5)	(20.5–24.7)
Rural	3617	(77.4)	(75.3–79.4)

Figures given in parentheses are weighted percentages

*CI* Confidence intervals

#### *Factors associated with non*-*utilization of ITN*

In 2015–2016, almost all households (97%) in Myanmar had at least one mosquito net (treated or untreated), but only 27% had at least one ITN (data not tabulated). Overall, 80.5% under-fives did not sleep under an ITN last night. In the adjusted analysis, malaria elimination regions (aOR = 2.0, 1.3–3.2, p-value = 0.003), urban residence (aOR = 1.8, 1.2–2.9, p-value = 0.006), residing in delta region (aOR = 8.7, 4.7–12.2, p-value < 0.001), hilly region (aOR = 3.0, 2.0–4.6, p-value < 0.001),and highest wealth quintile (aOR = 1.8, 1.1–3.0, p-value = 0.01) were significantly associated with non-utilization of ITNs (Table 3).

**Table 3:** Socio-demographic, geographic and household characteristics associated with non-utilization of ITNs among under-five children in Myanmar Demographic and Health Survey, 2015–16 (N = 4597)

Characteristics	Total	ITN non-utilization N (%)	Unadjusted OR (95% CI)	p-value	Adjusted OR (95% CI)	p-value
Total	4597	3328 (80)				
Age of the child (in months)				0.07		
36–59	1842	1355 (81)	1		1	
12–35	1815	1320 (81)	1.0 (0.8–1.2)		1.0 (0.8–1.2)	0.9
<12	940	653 (79)	0.9 (0.8–1.12)		0.9 (0.8–1.2)	0.5
Sex of the child				0.6		
Male	2402	1731 (80)	1			
Female	2195	1597 (81)	1.0 (0.9–1.2)			
Age of caregiver (in years)				0.8		
15–24	865	632 (81)	1			
25–34	2368	1715 (81)	1.0 (0.7–1.3)			
≥35	1364	981 (80)	0.9 (0.7–1.2)			
Education of the caregiver				< 0.001		
No formal education	802	539 (72)	1		1	
Primary	2034	1454 (80)	1.6 (1.1–2.3)		1.0 (0.7–1.4)	0.8
Secondary	1440	1058 (84)	2.0 (1.4–3.0)		1.0 (0.7–1.5)	0.9
More than secondary	321	277 (91)	3.9 (2.2–7.2)		1.3 (0.6–2.6)	0.5
Father’s education				< 0.001		
No education	792	532 (72)	1		1	
Primary	1797	1310 (82)	1.7 (1.2–2.5)		1.2 (0.8–1.7)	0.3
Secondary	1664	1208 (82)	1.8 (1.2–2.6)		0.9 (0.6–1.3)	0.5
More than secondary	253	212 (88)	2.9 (1.6–5.2)		0.8 (0.4–1.7)	0.6
Occupation of the caregiver				0.03		
Not-working	2170	1538 (80)	1		1	
Working	2425	1788 (81)	1.1 (0.9–1.4)		0.9 (0.7–1.2)	0.7
Father’s occupation				0.001		
Manual worker	2599	1892 (81)	1		1	
Agricultural	1280	886 (77)	0.8 (0.6–1.0)		0.8 (0.6–1.1)	0.1
Others	718	550 (83)	1.2 (0.8–1.6)		0.7 (0.5–1.1)	0.09
Number of household members				0.07		
1–3	450	344 (84)	1		1	
4–6	2606	1897 (81)	0.8 (0.6–1.2)		1.0 (0.7–1.5)	0.8
7–10	1335	938 (78)	0.7 (0.5–1.0)		1.1 (0.7–1.6)	0.7
More than 10	206	149 (78)	0.7 (0.4–1.3)		1.0 (0.5–2.2)	0.9
Wealth index	4,597			< 0.001		
Lowest	1383	928 (74)	1		1	
Second	1043	722 (77)	1.2 (0.9–1.6)		1.1 (0.8–1.4)	0.7
Middle	826	573 (81)	1.5 (1.1–2.2)		1.3 (0.9–1.8)	0.2
Fourth	775	616 (87)	2.4 (1.6–3.5)		1.8 (1.1–2.7)	0.01
Highest	570	489 (91)	3.4 (2.2–5.3)		1.8 (1.0–3.3)	0.06
Region				< 0.001		
Other regions	3100	2025 (73)	1		1	
Malaria elimination regions	1497	1303 (90)	3.4 (2.4–4.8)		2.0 (1.3–3.2)	0.003
Geographical location				< 0.001		
Coastal	895	459 (51)	1		1	
Hilly	1810	1174 (73)	2.6 (1.7–4.0)		3.0 (2.0–4.6)	< 0.001
Delta	810	764 (94)	14.9 (8.6–25.8)		12.7 (7.7–21.2)	< 0.001
Central plains	1082	931 (85)	5.3 (3.1–9.2)		4.0 (2.3–7.3)	< 0.001
Place of residence				< 0.001		
Rural	3617	2489 (77)	1		1	
Urban	980	839 (91)	3.1 (2.2– 4.6)		1.8 (1.2–2.9)	0.006

Figures presented here are weighted estimates

*ITN* insecticide treated net, *CI* Confidence Interval

#### Care-seeking for under-five children with a fever and factors associated with not seeking care

**Fig. 2:**
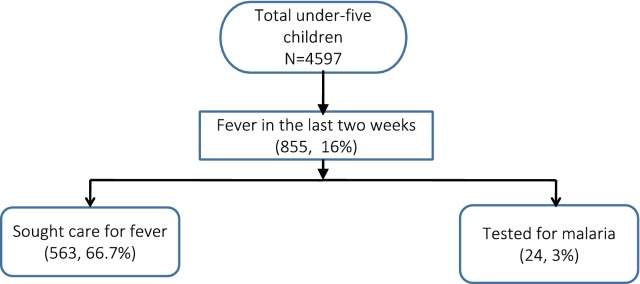
Fever among under-five children and uptake of care and testing services for fever among their caregivers in Myanmar Demographic and Health Survey, 2015–2016

Of 4597 under-five children, 16% (n = 855) had fever in the last 2 weeks, of whom 66.7% (n = 563) sought care for fever and 3% (n = 24) got tested for malaria (Fig. 2).

Overall, 33.3% did not seek care for fever at all in the last 2 weeks. In the adjusted analysis, geographical location (hilly, delta and central plains compared to coastal region) and lowest wealth quintile (aOR = 2.3, 1.1–5.7, p-value = 0.03), were significantly associated with not seeking care for fever (Table 4).

**Table 4:** Socio-demographic, geographic and household characteristics associated with non-seeking of care among caregivers of under-five children who had fever last 2 weeks in Myanmar Demographic and Health Survey, 2015–2016 (N = 855)

Characteristics	Total	Non-seeking of care N (%)	Unadjusted OR (95% CI)	p-value	Adjusted OR (95% CI)	p-value
Total	855	292 (33)				
Age of the child (in months)				0.1		
36–59	268	127 (35)	1		1	
12-35	411	64 (32)	0.9 (0.6–1.4)		0.9 (0.6–1.4)	0.6
<12	176	101 (35)	1.0 (0.6–1.7)		1.1 (0.6–1.9)	0.8
Sex of the child				0.8		
Male	440	152 (34)	1			
Female	415	140 (32)	0.9 (0.7–1.3)			
Age of caregiver (in years)				0.9		
15–24	178	58 (40 )	1			
25–34	428	148 (31)	0.7 (0.4–1.2)			
≥35	249	86 (31)	0.6 (0.4–1.2)			
Education of caregiver				0.1		
No education	149	54 (32)	1		1	
Primary	385	141 (36)	1.2 (0.6–2.3)		1.1 (0.6–2.0)	0.8
Secondary	275	88 (33)	1.1 (0.5–2.1)		1.2 (0.6–2.6)	0.6
More than secondary	46	9 (18)	0.5 (0.1–1.3)		0.7 (0.2–2.1)	0.5
Father’s education				0.61		
No education	134	45 (29)	1			
Primary	357	127 (37)	1.5 (0.8–2.7)			
Secondary	309	100 (30)	1.0 (0.5–2.0)			
More than secondary	35	9 (25)	0.8 (0.3–2.5)			
Occupation of caregiver				0.07		
Not Working	372	115 (30)	1		1	
Working	481	177 (36)	1.3 (0.9–1.9)		1.2 (0.8–1.7)	0.8
Father’s occupation				< 0.001		
Manual worker	477	131 (32)	1		1	
Agricultural	266	125 (39)	1.4 (0.9–2.2)		1.2 (0.8–1.9)	0.4
Others	112	36 (29)	0.9 (0.5–1.6)		1.1 (0.5–2.2)	0.8
No of household members				0.7		
1–3	67	19 (30)	1			
4–6	466	159 (32)	1.1 (0.6–2.2)			
7–10	271	96 (35)	1.3 (0.6–2.5)			
More than 10	51	18 (41)	1.7 (0.6–4.7)			
Wealth index				< 0.001		
Highest	86	19 (23)	1		1	
Fourth	137	29 (23)	1.0 (0.5–1.9)		0.9 (0.4–1.8)	0.7
Middle	128	50 (42)	2.3 (1.1–5.1)		2.3 (1.0–5.1)	0.04
Second	226	87 (34)	1.7 (0.8–3.6)		1.7 (0.7–3.9)	0.2
Lowest	278	107 (38)	2.0 (0.9–4.1)		2.3 (0.9–5.7)	0.08
Region				0.6		
Other regions	654	220 (32)	1			
Malaria elimination region	201	72 (37)	1.2 (0.8–2.0)			
Geographical location				< 0.001		
Coastal	169	31 (18)	1		1	
Hilly	403	158 (37)	2.7 (1.4–4.9)		2.8 (1.5–5.3)	0.002
Delta	144	50 (34)	2.3 (1.2-4.4)		2.3 (1.–4.6)	0.01
Central plains	139	53 (38)	2.8 (1.4–5.3)		3.1 (1.5–6.2)	0.002
Place of residence				0.3		
Rural	683	239 (33)	1			
Urban	172	53 (34)	1.1 (0.6–1.8)			

Figures presented here are weighted estimates

CI = Confidence Interval

#### *First treatment*-*seeking source for fever*

**Fig. 3:**
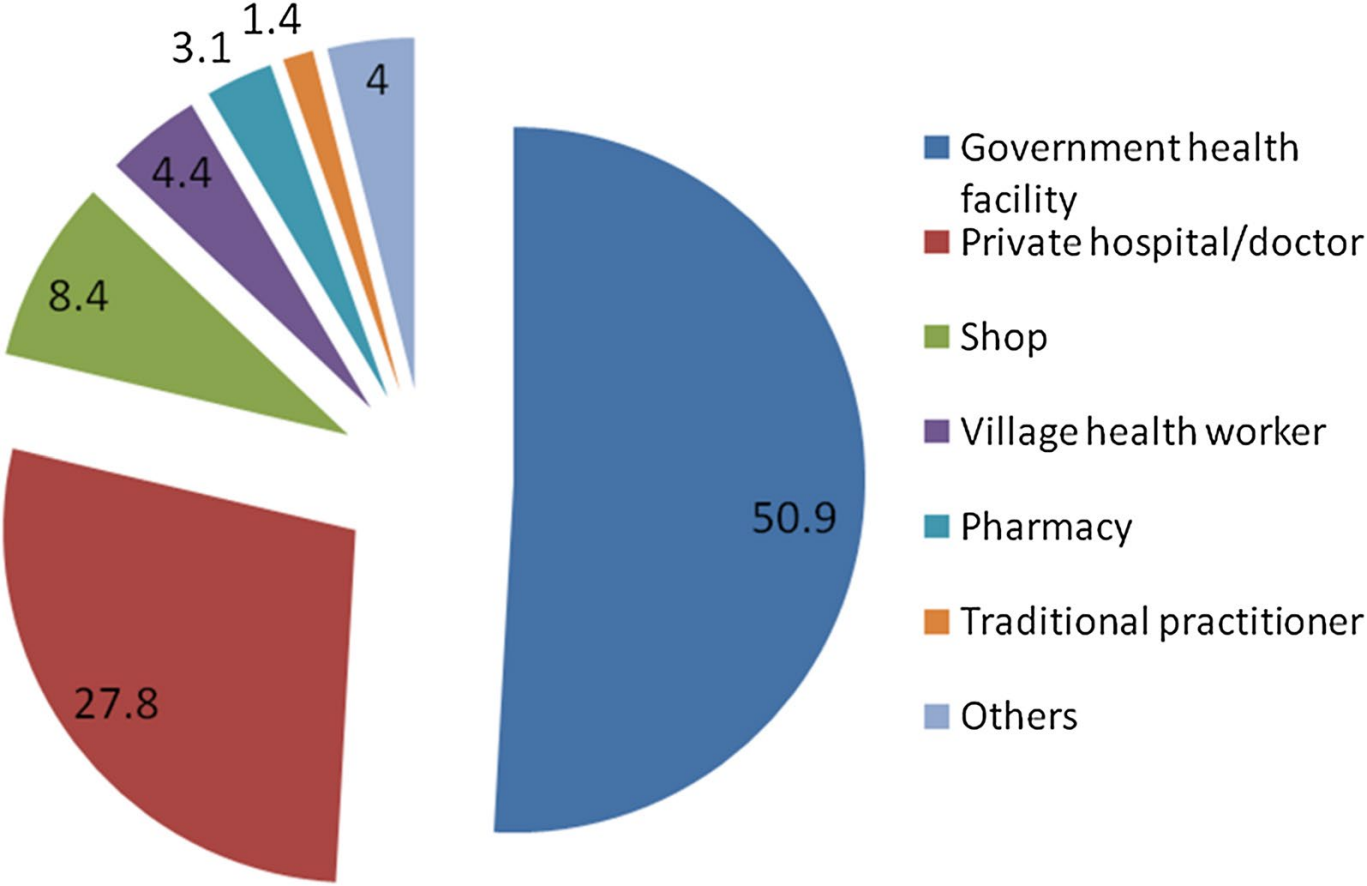
Type of first care provider sought by caregivers of under-five children with fever in the Myanmar Demographic and Health Survey, 2015–2016 (n = 563)

Around half (50.9%) of the caregivers sought care from a government health facility, followed by private hospital/doctor (27.8%), shop (8.0%), village health worker (4.4%) and pharmacy (3.1%) (Fig. 3).

## Discussion

This is the first nationally representative study from Myanmar reporting care-seeking behaviour for fever and ITN utilization among the vulnerable under-five population. The study had some interesting findings.

This study finding highlighted that only less than one-fifth of under-five children slept under an ITN the night prior to the survey. This is probably due to poor ownership of ITNs which is also reported in previous studies from Myanmar [12, 15]. This calls for mass distribution of ITNs with emphasis on households with under five children to improve ownership of ITNs. Household mapping which is done before distribution of ITNs should be meticulously planned especially while doing a head count in households with under five children.

Previous studies in African countries have reported higher utilization of ITNs among children ranging from 42 to 51% [7, 23]. Studies from Myanmar have also reported higher ITN utilization of 45–50% among the migrant population and the Regional Artemisinin Resistance Initiative areas of Myanmar, probably due to the increased focus of the programme through heightened routine activities and increased external funding in these high risk areas [24, 25]. Reasons for poor ITN utilization among caregivers of under-five children in this study need to be explored through qualitative research.

This study demonstrated higher utilization rates for ITN among rural children than their urban counterparts similar to previous studies [26]. This was contrary to what was found in a meta-analysis of 13 surveys, which included five Demographic Health Surveys for African countries, where children in urban households were found to be more likely to use nets than children in rural households [27]. One possible reason for this could be the low incidence of malaria in urban areas such as Yangon and Mandalay which are moving towards malaria elimination. This leads to low perceived threat of mosquito bite and probably also explains the poor ITN utilization in malaria elimination regions. However, the exact reasons require an in-depth qualitative exploration.

Low ITN use has been reported in the delta and hilly regions. This is concerning for the programme as these are high malaria transmission regions. The reasons are beyond the scope of this study, warranting further qualitative studies.

Among the caregivers of under-five children with a fever, only two-third sought care. This figure is much lower than the 100% defined by the Myanmar National Malaria Control Programme [28]. This finding is alarming, given that malaria is a major cause of fever in children in Myanmar and prompt care-seeking is necessary to reduce morbidity and mortality. It suggests the need for intensified social and behaviour change communication strategies to improve care-seeking efforts. The results are similar to what was reported in previous studies from Senegal and Mozambique, in which around one-third of children with fever did not receive any treatment or medical advice [29, 30].

However, previous studies from Myanmar have reported higher proportion (~ 80%) of children with fever receiving care, possibly because these studies were conducted in specific groups of populations such as the migrants, ethnic minority groups and malaria endemic rural areas which are high priority areas for the national malaria programme and receives maximum attention and resource allocation. Also, the definition used to define care-seeking varied across studies [21, 31, 32].

Respondents residing in the hilly areas have reported poor care-seeking probably due to the difficult terrain, thus creating access barriers to seeking care. The role of village level volunteers is crucial in these areas to provide doorstep decentralized services.

This study showed poor care-seeking among caregivers of under-five children belonging to the lowest socio-economic status. This has been reported by several studies from low-middle income countries [33–35]. These findings are also consistent with studies focusing on other childhood illnesses, such as diarrhoea and ARI in similar settings [36, 37]. Concerns about payment for consultation remain a barrier to seeking health care and achieving better health outcomes for the poor.

The proportion of children with fever getting tested for malaria was dismally low with only 3%. Similar concerns were also raised in previous studies among migrants and the general population in Myanmar which reported lower uptake of malaria testing (12–24%) [18, 32]. Several patient (self-medication, not giving due importance to fever, transportation difficulty, uninformed about malaria testing by VHV) and provider (lack of test kits, testing only seriously ill patients) related factors were identified in a qualitative study done previously to explain poor uptake of malaria testing [32]. These factors need to be tackled as part of a comprehensive strategy to improve the knowledge and practice of caregivers related to malaria, especially the need for testing within 24 h of fever. Malaria being a major killer among under-fives, appropriate management of fever among children should be a national programme priority to achieve zero malaria deaths.

The major strengths of the study were that the data were obtained from a large nationally representative survey covering all the states/regions of Myanmar; the survey followed a standard DHS methodology; the response rate was high; missing values were minimum; and the subject is an identified national and global research priority. The study adhered to the Strengthening the Reporting of Observational Studies in Epidemiology (STROBE) guidelines for the reporting of observational studies.

The study had few limitations. The study did not explore reasons for poor health-seeking behaviour in case of fever and poor utilization of ITNs which require qualitative studies in future. Also, the study did not explore other personal and behavioural factors such as knowledge, attitude, beliefs and perception regarding the role of ITN and health care-seeking which are known to influence behaviours. The responses were self-reported which could be influenced by social desirability bias, although, wherever possible, observation of self-reported ITNs and ITNs mounted over the sleeping area was done during the interview.

## Conclusion

This study highlighted poor ownership and utilization of ITNs among the under five children. Nearly one-third of the caregivers of under-five children did not seek care for fever which has implications on childhood morbidity and mortality. The programme should seriously consider addressing these barriers if Myanmar is to achieve zero malaria deaths by 2030. Continuous and strengthened community awareness campaigns in various forums such as immunization/antenatal sessions, under five clinics, and other community gatherings is needed to bring about appropriate change in behaviour.

## Data Availability

Data are not available in public domain because they are currently being analyzed in related papers. However, data are available with the corresponding author (KTM) and will be made accessible on reasonable request at the following e-mail: drktmin@gmial.com.

## Abbreviations

3MDG: three millennium development goals fund
DMR: Department of Medical Research
DHS: Demographic and Health Survey
RHC: rural health centre
UHC: urban health centre
MOHS: Ministry of Health and Sports
FGD: focus group discussion
BHS: basic health staff
GMS: greater Mekong subregion
ITN: insecticide-treated bed net
LLIN: long-lasting insecticidal net
RDT: rapid diagnostic test
NMCP: National malaria control programme
RAI: regional artemisinin initiative
WHO: World Health Organization
